# Acknowledgment to Reviewers of *Pharmaceutics* in 2020

**DOI:** 10.3390/pharmaceutics13020154

**Published:** 2021-01-25

**Authors:** Pharmaceutics Editorial Office

**Affiliations:** MDPI AG, St. Alban-Anlage 66, 4052 Basel, Switzerland

Peer review is the driving force of journal development, and reviewers are gatekeepers who ensure that *Pharmaceutics* maintains its standards for the high quality of its published papers. Thanks to the cooperation of our reviewers, in 2020, the median time to first decision was 14 days and the median time to publication was 35 days. The editors would like to express their sincere gratitude to the following reviewers for their precious time and dedication, regardless of whether the papers were finally published:
Abakumov, Maxim A.Liu, Yun-JunAbatzoglou, NicolasLiu, ZhiAbbina, SrinivasLobo, Glenn P.Abd El Hakim, YasminaLochhead, Jeffrey J.Abdel-Daim, MohamedLollo, GiovannaAbdeltawab, NourtanLong, Chiau MingAbejón, RicardoLongo, Joao PauloAbraham, Wolf-RainerLönnberg, HarriAbruzzo, AngelaLopalco, AntonioAcedo Nunez, Maria Del PilarLopedota, AngelaAcharya, GhanashyamLopes, Carla MartinsAcri, GiuseppeLópez Castellano, AliciaAdebisi, Adeola O.Lopez-Camarillo, CesarAderibigbe, BlessingLópez-Camarillo, CésarAderibigbe, Blessing Atim A.Lopez-Cara, Luisa CarlotaAdil, Syed FarooqLópez-Cornejo, PilarAdobes-Vidal, MariaLópez-López, ManuelAdorni, Maria PiaLorberboum-Galski, HayaAdrover, AlessandraLoretz, BrigittaAdunyah, Samuel E.Loureiro, Joana A.Afshari, KhashayarLozano, Jose ManuelAfzal, AdeelLu, MingAgatemor, ChristianLu, WeiyueAgrahari, VivekLu, YiAguirre, AitorLucaciu, Constantin MihaiAguirre-Álvarez, GabrielLucas, Andrew T.Ahad, AbdulLucas, GuilhermeAhmad, AliLucchetti, DonatellaAhmad, JavedLuciani, PaolaAhmad, ZeeshanLucio, VictorAhmed, MaryaLuckham, PaulAhmed, Osama A. A.Luebbert, ChristianAigner, ZoltánLugović-Mihić, LiborijaAiraksinen, Anu J.Lukyanov, Pavel A.Aitipamula, SrinivasuluLuliński, PiotrAkagi, YukiLunter, DominiqueAkhlaghi, Seyedeh ParinazLuo, KuiAkita, SadanoriLuo, RongcongAl Qahtani, MohammedLuppi, BarbaraAlanis-Lobato, GregorioLusi, MatteoAlavi, Seyed EbrahimLux, JacquesAlbaladejo Vicente, RomanaLuzardo-Álvarez, AsteriaAlbericio, FernandoLuzi, FrancescaAlbrechtsen, Nicolai J. WewerM. Caramella, CarlaAlcudia, AnaMa, JunAldred, KatieMa, XiangAleandri, SimoneMaayah, ZaidAlegret, NuriaMachado-Aranda, DavidAlessandro, CecconelloMachelart, ArnaudAlexander, CameronMadheswaran, ThiagarajanAlexa-Stratulat, TeodoraMadhyastha, HarishkumarAl-Fatimi, MohamedMadka, VenkateshwarAlfei, SilvanaMaeda, KazuyaAlghamdi, Hamdan S.Maeda, TomokiAl-Gousous, JozefMaeng, Han-JooAlhakamy, NabilMaestrelli, FrancescaAlhareth, KhairMaggio, Rubén M.Ali, Adel AMagnani, MauroAljayyoussi, GhaithMagri, GiuliaAlkilani, Ahlam ZaidMaier, Jeanette A.MAllegaert, KarelMair, Lamar O.Allen, Sean DavidMaisel, KatharinaAlmoazen, HassanMajkowska-Pilip, AgnieszkaAlmoazen, HassenMajolino, DomenicoAlonso-Moreno, CarlosMajor, IanAlsaab, HashemMajumder, AniruddhaAl-Tabakha, Moawia M.Majumder, PoulamiAltmann, FriedrichMakarska-Bialokoz, MagdalenaAltucci, LuciaMalcolm, KarlAlves, CarlosMaleki, HajarAlves, FranciscoMalik, AdeelAmbrus, RitaMalik, Paul R. V.An, YuMalik, RavinderAnanthakrishnan, Soundaram JeevarathinamMalinge, Jean-MarcAndo, KojiMallandrich, MireiaAndrić, FilipMalo De Molina, PaulaAndriolo, JessicaMalzert-Fréon, AurélieAngela Barba, AnnaMamidi, NarmidiAngelopoulou, AthinaManakhov, AntonAngelov, BorislavManca, Maria LetiziaAngelova, AngelinaManconi, MariaAngelucci, EmanueleMandal, AbhirupAngsantikul, PavimolManetti, DinaAnne-Claire, GrooManfredini, StefanoAnraku, MakotoMangal, SharadAnsari, D. Mohd. AzamMangas, VictorAnsari, Mohammad JavedMangino, GiorgioAnsorena, EduardoManiruzzaman, MohammedAntal, IstvánManna, SaikatAntimisiaris, Sophia G.Mansi, RosalbaAntipina, Maria N.Mao, QingchengAntoniac, IulianMarcos-Contreras, Oscar AAntoniu, Sabina AntonelaMarei, HanyAnzenbacherova, EvaMareș, MihaiAparicio-Blanco, JuanMargreitter, ChristianApostolopoulos, VassoMarín Ramos, Nagore IsabelAquavella, James V.Marin, LuminitaAran, VeronicaMarino Gammazza, AntonellaArana, LideMarkl, DanielAraújo, Thaíse GonçalvesMarkowicz-Piasecka, MagdalenaAriatti, MarioMaroni, AlessandraAriga, KatsuhikoMarová, IvanaArmakovic, Sanja J.Marradi, MarcoArmanini, DecioMartellucci, StefanoArnolds, HeikeMartín Rengel, Antonio E.Arufe, María CMartin, FranciscoArunachalam, PrabhakarnMartin, Maria AlbaAshihara, EishiMartín-Aragón, SagrarioAshley, JonMartinez Alonso, MartaAso, YoshimasaMartinez Rodriguez, FlemingAugustyniak, AdrianMartinez, CristinaAungst, BruceMartinez, VicenteAvgoustakis, KonstantinosMartínez-Carmona, MarinaAwasthi, VibhuduttaMartínez-García, MarcosAzam, FaizulMartínez-López, Ana LuisaAzer, Samy A.Martino, SabataAzevedo, Nuno F.Martins, Alessandro F.Azzimonti, BarbaraMartins, José A.Babič, MichalMartins, NatáliaBabincová, MelániaMarto, JoanaBaby, André RolimMarverti, GaetanoBacci, StefanoMarwitz, SebastianBach, HoracioMarzano, CristinaBácskay, IldikóMasalova, Olga V.Badea, IldikoMas-Bargues, CristinaBadea, NicoletaMashima, RyuichiBadimon, Juan J.Mashruwala, AmeyaBado, IgorMaslov, Mikhail A.Badr, GamalMassa, AntonioBae, JeehyeonMastinu, AndreaBaek, In-HwanMastrotto, FrancescaBaer, Patrick C.Masuda, TakeshiBagyinszky, EvaMatei, CristianBahal, RamanMatencio, AdriánBahn, AndrewMatijašić, GordanaBai, ShiMatikonda, SiddharthBai, ShuhuaMATRICARDI, PietroBaietti, Maria FrancescaMatsugaki, AiraBajek, AnnaMatsui, ReikoBakowsky, UdoMatsumoto, YasuhikoBalalaeva, Irina V.Matsunaga, KazuhisaBalanč, BojanaMatsuura, BunzoBalasubramanian, KrishnanMatta, Murali KrishnaBalcerczyk, AnetaMattei, VincenzoBallard, DavidMatters, Gail L.Banciu, ManuelaMatthiesen, RuneBandarenka, Hanna V.Matys, JacekBandari, SureshMauri, EmanueleBandyopadhyay, SulalitMayer, ChristianBansal, KuldeepMazel, VincentBansal, Kuldeep K.Mazet, RoselineBanti, Christina N.Mazzaglia, AntoninoBao, YinyinMazzoni, ChiaraBao, YongmingMcardle, PatrickBaran, AnnaMcArdle, StephanieBarbalexis, PanagiotisMcCartney, FionaBarbosa, Carlos MauricioMcCauley, Heather ABarbosa, Raquel De MeloMcConville, ChristopherBarca, AmilcareMcConville, Jason T.Barelle, CarolineMcdonald, PeterBari, EliaMcDowell, ArleneBarlow, DaveMcHugh, KevinBarolo, MassimilianoMcMurry, JonathanBarra, GiusiMd, ShadabBarradas, Thaís NogueiraMedarevic, DjordjeBarrett, Robert J.Medeiros, Ana Cláudia D.Basit, AbdulMedina Juarez, Luis AngelBaswan, SudhirMedina Velázquez, Luis AlbertoBatchelor, Hannah KatharineMedina-Ramírez, IlianaBattaglia, LuigiMees, MaartenBauckneht, MatteoMei, Ya-FangBax, Bridget E.Meier, Robert J.Bayer, WibkeMelero, AnaBazrafshan, ZahraMelo-Diogo, Duarte DeBazwinsky-Wutschke, IvonneMendonça-Lima, LeilaBazylinska, UrszulaMendoza-Muñoz, NéstorBegines Ruiz, BelénMendyk, AleksanderBegines, BelenMenghini, LuigiBello, Nicholas T.Merino, SoniaBelvedere, RaffaellaMerlino, FrancescoBenhabbour, Soumya RahimaMestres, ConxitaBenito, MónicaMeyer, FlorentBenny, OfraMező, GáborBenson, HeatherMezzetta, AndreaBerger, Daniela CristinaMi, Fwu-LongBerger, GillesMi, LanBergonzi, Maria CamillaMicek, AgnieszkaBerkó, SzilviaMielańczyk, AnnaBermejo, MarivalMihai, MaraBernal, EvaMijovic, BudimirBernal, Freddy AlexanderMilasius, RimvydasBernardi, SimonaMilavetz, BarryBerthier, CelineMilazzo, MarioBessa, LucindaMildner, MichaelBettache, NadirMilewska, Maria JBettini, RuggeroMiller, Ian SteuartBeuran, MirceaMiller, LarryBhargava, AditiMinami, MasaakiBhatt, PriyankaMinghetti, PaolaBiagi, MarcoMircioiu, ConstantinBiagini Lopes, LucianaMirza, SabiruddinBiagini, GiuseppeMishra, Pawan KumarBianco, FedericoMitchell, AndrewBidarra, Sílvia J.Miura, YoshikoBidwell, GeneMiyake, MasateruBiernasiuk, AnnaMiyata, YoshihikoBilgili, EcevitMiyata, YoshikiBilia, Anna RitaMiyoshi, NorioBillotey, ClaireMlera, LuwanikaBinda, ElisaMochizuki, ShinichiBini, MarcellaModi, NisargBins, Adriaan D.Mohammed, YousufBiondi, MarcoMoiseev, Roman V.Birnbaum, Angela K.Mollapour, MehdiBlaabjerg, LasseMomin, Mohammad Abdul MotalibBlagbrough, Ian S.Mondal, GoutamBlanco-Fernandez, BarbaraMondal, SudipBlanco-López, María CarmenMontanari, ElitaBłażewicz, AnnaMonteiro, CarlosBlower, Philip J.Monteiro, CláudiaBobbala, SharanMontenegro, LuciaBobis, OtiliaMonti, DanielaBobis-Wozowicz, SylwiaMoon, CheolBoddu, Sai HanumasagarMoon, Geon DaeBodea, Liviu-GabrielMooranian, ArminBodenlenz, ManfredMora, ClaudiaBoekema, BoukeMorales, JavierBogdan, CatalinaMorales-Rojas, HugoBoggio, ElenaMorbidelli, LuciaBoix-Montañés, AntonioMoreau, RegisBoldyreva, ElenaMorefield, GarryBollati, MarielaMorgado, JorgeBombieri, CristinaMorgat, ClémentBonadies, IreneMorgen, MichaelBonam, Srinivasa ReddyMoritz, MichałBoncoeur, EmilieMornar, AnaBonferoni, Maria CristinaMorotomi, TakahikoBono, NinaMorris, GordonBony, Badrul AlamMorris, John B.Borrego-Sánchez, AnaMosqueira, MatiasBorska, SylwiaMosqueira, Vanessa Carla FurtadoBorst, PietMoss, GaryBosland, Maarten C.Mourtas, SpyridonBosquillon, CynthiaMouzakis, Dionysios E.Botton, ThomasMoyano-Mendez, Jose RamonBouchecareilh, MarionMracek, TomasBoudon, JulienMu, QingxinBoudreau, Paul D.Muchowicz, AngelikaBourdon, EmmanuelMuchtaridi, MuchtaridiBouropoulos, NikolaosMuenzberg, MichaelBouyer, FredericMujtaba, MuhammadBoyd, Ben J.Mukherjee, DwaipayanBoyd, ChrisMukherjee, RajBraconi, DanielaMukherjee, SudipBradley, Laura C.Muller, ChristianBraeuer, AndreasMundra, VaibhavBrako, FrancisMuntean, Dana MariaBrayden, David J.Muntimadugu, EameemaBrea-Calvo, GloriaMurab, SumitBreier, AlbertMurakami, TeruoBreitkreutz, JörgMurnane, KevinBrenišin, MarekMusazzi, Umberto M.Brenza, TimothyMusuc, Adina MagdalenaBriançon, StéphanieMusumeci, DomenicaBrignole, ChiaraMusumeci, TeresaBroer, StefanMuthu Karuppan, Mohan KumarBroude, EugeniaMuthuraju, SanguBrouillet, EmmanuelMuthusamy, NagendranBrown, Mark A.Muzykantov, Vladimir R.Brozovic, AnamariaMuzzalupo, RitaBrozyna, AnnaMyc, AndrzejBruni, GiovannaMyers, Alan LewisBruno, AntoninoNa, Dong HeeBryant, Christian E.Na, Young-GukBrzeska, JoannaNag, Okhil K.Brzezinski, MarekNagai, NoriakiBrzozowski, TomaszNagata, ToshiBudai-Szűcs, MáriaNair, Anroop B.Bueno-Silva, BrunoNair, Devatha P.Buj-Corral, IreneNair, Praful R.Bukowska, JoannaNair, PramodBulatovic, MajaNajlah, MohammadBunt, CraigNakai, HiroyukiBurckart, Gilbert JNakamura, KazufumiBurgalassi, SusiNakanishi, TakeoBurley, Jonathan C.Nakano, MasahitoBusco, GiovanniNakielski, PawelBüsselberg, DietrichNAM, Ki ChangCaballero-Casero, NoeliaNamgaladze, DmitryCacciatore, IvanaNamjoshi, SarikaCadinoiu, Anca NiculinaNanaki, StavroulaCaenazzo, LucianaNanavati, CharviCai, ChaoNanjundan, MeeraCai, LishengNarayan, MaheshCai, WeiminNarayanasamy, SureshCaldera, FabrizioNarayanaswami, VasanthyCalvignac, BriceNartowski, Karol P.Cameron, DonaldNatesan, ShanmugasundaramCaminade, Anne-MarieNavarrete, PaolaCammas-Marion, SandrineNavarro, EstanisCampana, StefaniaNavarro-Ruiz, AndrésCampos Rosa, Joaquín MaríaNavran, ArashCanals, FrancescNawaz, MuhammadCanfield, ScottNayak, Usha Y.Cano, AmandaNaylor, AndrewCantore, AlessioNegri, DonatellaCao, XudongNethi, Susheel KumarCapasso, RaffaeleNeubert, ReinhardCapogni, MarcoNeufurth, MeikCapozzi, Luigi CarloNeves, VeraCaputo, GregoryNg, Keng WooiCarafa, MariaNgen, Ethel J.Caramella, Carla MarcellaNi, RongCarbone, ClaudiaNicolau, DavidCarmona-Ribeiro, Ana M.Nicoli, SaraCarpes, Solange TeresinhaNielsen, Line HagnerCarrasco, ElisaNiemczyk, AgataCarrotta, RitaNiemirowicz-Laskowska, KatarzynaCarter, KatharineNightingale, AdrianCaruntu, ConstantinNikolakakis, IoannisCasalini, TommasoNikolett, KállaiCasas-Solvas, Juan M.Nishida, KoyoCasiraghi, AntonellaNita, Loredana E.Cassano, DomenicoNoh, KeumhanCasteleijn, MarcoNokhodchi, AliCastorena-Torres, FabiolaNoma, HidetakaCastro, Ricardo A.E.Nopens, IngmarCatalin, BogdanNosoudi, NasimCatanzano, OvidioNotario-Pérez, FernandoCatenacci, LauraNovikov, AndreiCatucci, LuciaNovinec, MarkoCauda, ValentinaNsooudi, Nasim NsooudiCausin, PaolaNumez, EstrellaCefalas, Alciviadis-ConstantinosNurunnabi, MdCelakovska, JarmilaNuxoll, EricCelebioglu, AsliO’Connor, José EnriqueCelia, ChristianO’Shaughnessy, RyanCemazar, MajaObata, YasukoCeña, ValentínObeng, SamuelCenciarelli, CarloObuobi, Sybil Akua OkyerewaČerný, JiříOchowiak, MarekCerreta, FrancescaOgawa, KazumaChacko, Jenu VargheseOh, DanielChae, Jung-wooOh, In-JaeChakraborty, AtanuOh, Ji-HyeonChallagundla, KishoreOh, Kyung TaekChamcheu, Jean ChristopherOjha, Shreesh K.Chan, Hon FaiOka, SarangChan, Shih-HsuanOkamoto, MotokiChandasana, HardikOkamura, YosukeChandrudu, SaranyaOkesola, BabatundeChang, Ching-ChihOkuda, TomoyukiChanishvili, NinaOlaru, Octavian TudorelChaput, FrédéricOlaso, GloriaChaud, MarcoOlczyk, PawełChaudhari, SmrutiOlewnik-Kruszkowska, EwaChauhan, NeerajOliveira, David DeChavan, HemantkumarOliveira, J.VladimirChen, DaiqinOltolina, FrancescaChen, HaoOmasa, TakeshiChen, HaoqingOmote, HiroshiChen, Jyh-PingOnishi, HirakuChen, Kelvin H.-C.Ordaz-Ortiz, José JuanChen, Kuo-YuOrlando, TomasChen, LinOrlova, AnnaChen, Mei-ChinOrtega, PaulaChen, MingshengOsada-Oka, MayukoChen, MingzhouOstacolo, CarmineChen, PeilinØstergaard, JesperChen, QixianOstos Marcos, Francisco JoséChen, XinyuanOstrovsky, OlgaChen, Yeong-RennOswald, StefanChen, Yuan-ChuanOsyczka, Anna MariaCheng, Hung-ChiOta, SatoshiCheng, Jya-WeiOtero, CarolinaChereddy, KiranOtero-Espinar, Francisco JavierChetoni, PatriziaOtsuka, MakotoChiang, Chih-ShengOtte, Andrew D.Chiang, Po-ChangOtto, Daniel P.Chiang, Yi-TingOuro, AlbertoChien, ChianshiuOuyang, DefangChiesa, EnricaOuyang, Xiao-kunChiriac, AuricaOya, YuichiCho, Cheong-WeonOzawa, ShogoCho, Dong-WooOzsvari, BelaCho, Hea-YoungPacifici, RobertaCho, HyunahPadrela, LuisCho, Hyun-JongPaduch, RomanChoi, JonghoonPagano, CinziaChoi, Seok KiPage, SusanneChong, LiPaine, StuartChong, Parskson Lee GauPaixão, PauloChoquesillo-Lazarte, DuanePal, DhananjayChou, Shih-FengPalamà, IlariaChow, Aviva Shing FungPall, EmokeChow, JamesPalmiero, Umberto CapassoChow, Michael Y.T.Palugan, LucaChowdhry, Dr B. Z.Pan, GuoqinChowdhury, Ezharul HoquePan, GuoqingChrist, BrunoPan, GuoyuChrobak, ElwiraPan, JiayiChuang, Er-YuanPanayiotou, CostasChuang, Yu-ChunPanczyk, TomaszChung, Suk-JaePandey, Amit VChwalibóg, AndréPangeni, RudraChworos, ArkadiuszPanikkanvalappil, Sajanlal R.Ciaccio, MarcelloPant, BishweshwarCiarimboli, GiulianoPanzarini, ElisaCieśla, JolantaPaolino, DonatellaCifuentes-Rius, AnnaPapadopoulos, DimitriosCimas, Francisco J.Papadopoulou, LefkotheaCipak, LubosPapakyriakou, AthanasiosCiszewski, Wojciech MichałPapini, EmanueleCiura, KrzesimirPapke, Roger L.Clares, BeatrizPaquette, BenoitCobos-Puc, LuisParamythiotou, ElizabethCoburn, JeannineParashar, DeepakCoclite, Anna MariaParayath, NehaCodacci-Pisanelli, GiovanniParayath, Neha N.Coelho, Sílvia CastroPardi, NorbertCohen, GuyParis, JuanCollado-González, MarPark, ChulhunCollins, MauricePark, FrankColombo, GaiaPark, Hwan-WooColombo, PaoloPark, Jeong-SookCometa, StefaniaPark, Jun BeomComi, CristoforoPark, Jung-HwanConcheiro, AngelPark, Ki SooConejos-Sánchez, InmaculadaPark, KinamConstantinescu, Cristina AnaPark, Kyung MinConti, BicePark, WooramConway, BarbaraParojčić, JelenaCooley, Brian C.Parrish, RichardCorbi-Verge, CarlesPassaes, Caroline Pereira BittencourtCorbo, ClaudiaPasserini, NadiaCordeiro, Ana SaraPastor, FernandoCordeiro, Rosemeyre AmaralPastor-Anglada, MarçalCórdoba, ManuelPatel, KetanCórdoba-Diaz, ManuelPatil, VeerupaxagoudaCorvis, YohannPatterson, Adam V.Costa-Almeida, RaquelPatterson, JenniferCostabile, GabriellaPaudel, RajCostello, JamesPaulusse, JosCote, ChristopherPautz, AndreaCourtenay, AaronPavliňáková, VeronikaCoutinho, PaulaPavlos, KlepetsanisCraig, DuncanPavlovic, NebojsaCristiano, ChiaraPawlik, AndrzejCroft, SimonPedrosa, PedroCrucho, CarinaPeeters, ElisabethCrucho, Carina Isabel CorreiaPeeters, MarloesCruz, Juan C.Peinado, María ÁngelesCsapo, EditPeláez, RafaelCsiszár, AgnesPellegatta, SerenaCsóka, IldikóPeltonen, LeenaCsordas, AndrewPeluso, IlariaCulot, MaximePeña Fernández, María ÁngelesCunha-Filho, MarcilioPenchev, HristoCurtis, Anthony D.M.Penfornis, PatriceCurtis, RobinPeng, LihongCysewska, KarolinaPeptu, CristianCzapik, AgnieszkaPerazzoli, GloriaD. Savić, SnežanaPerche, FedericoD’Amora, UgoPerche, FédéricoD’Apice, LucianaPerduca, MassimilianoDa Pieve, ChiaraPereira, RubenDa Silva Júnior, Arnóbio AntonioPereira-Leite, CatarinaDa Silva, A. MoreiraPérez De Val, BernatDa Silva, Luis Cláudio NascimentoPerez-Alvarez, L.Da Silva, Luís PintoPérez-Gracia, María-TeresaDagmar, MerinskaPerinelli, Diego RomanoDahlgren, DavidPerioli, LuanaDai, ChongshanPeris, José-EstebanDal Piaz, FabrizioPerissutti, BeatriceDalhaimer, PaulPerrone, Anna MyriamDalmoro, AnnalisaPerteghella, SaraDandawate, PrasadPerugini, PaolaDangol, ManitaPeruzynska, MagdalenaDaniela, AilincaiPetcu, CristianDaniels, RolfPetrova, GuenkaDanielsen, MichaelPetrović, LidijaDansette, Patrick M.Peviani, MarcoDargaville, TimPiacentini, EmmaDarvin, MaximPiao, XijunDas, SudipPiatkevich, KirylDash, Ranjeet PrasadPiato, AngeloDate, AbhijitPiedade, Ana PaulaDatta, KausikPierdomenico, Johnny DiDavalos, Rafael VPilicheva, BisseraDave, RuteshPilli, NageswaraDavid, Sheba R.Piluzza, GiannellaDe Araújo, Daniele RibeiroPindelska, EdytaDe Bock, MarijkePinheiro, LídiaDe Caro, VivianaPini, Luigi AlbertoDe Carvalho, Carla C. C. R.Pinnapireddy, Shashank ReddyDe Kruijff, Robin M.Pinteala, MarianaDe La Mata, JavierPinto, EugéniaDe Luca, MichelePinto, JoaoDe Melo-Diogo, DuartePinto, SandraDe Miguel, DiegoPiquette-Miller, MichelineDe Oliveira, Paulo RenatoPiras, Anna MariaDe Pauw, EdwinPisaneschi, FedericaDe Rosa, EnricaPiscioneri, AntonellaDe Rosa, GiuseppePishnamazi, MahboubehDe Sousa, Célia TavaresPissarek, MargitDe Sousa, Frederico B.Pitsikalis, MarinosDe Villiers, Melgardt M.Pitt, KendalDe, RanjitPitterl, FlorianDe, TanimaPitto-Barry, AnaïsDea-Ayuela, María AuxiliadoraPiva, TerrenceDegoul, FrançoisePlaninšek, OdonDelgado, AraceliPłaza, GrażynaDelgado, Daniel RicardoPloetz, EvelynDelgado-Charro, M. BegoñaPlotniece, AivaDeli, Mária A.Poeggeler, BurkhardDémuth, BalázsPoggi, AlessandroDen Haan, Joke M.M.Pohl, Carolina H.Desai, DitiPohlmann, AdrianaDesando, GiovannaPokorny, MarekDey, DebayanPolakowski, Nicholas J.Dhenge, RanjitPolgarova, KamilaDhilip Kumar, Sathish SundarPollet, JeroenDi Girolamo, MariaPolo-Parada, LuisDi Liegro, ItaliaPolyakov, NikolayDi Maro, AntimoPoma, AlessandroDi Martino, AntonioPomel, SebastienDi Martino, PieraPopa, LacramioaraDi Paolo, AntonelloPopat, AmiraliDi Pasqua, Anthony J.Porollo, AlekseyDi, LiPorter, Christopher J. H.Dias, Marcos LopesPoŝa, MihaljDiat, OlivierPost, JanineDigiacomo, MariaPotrich, CristinaDijk, FrederikePouliopoulos, AntonisDillman, Robert O.Pourbaghi Masouleh, MiladDimitrov, IvayloPrabha, SwayamDina, Nicoleta ElenaPradhan, ShankaliDing, JianxunPramod, KannisseryDinica, Rodica-MihaelaPranjol, ZahidDinu-Pirvu, Cristina-ElenaPrashar, SanjivDioguardi, MarioPrassl, RuthDireito, RosaPrat, MariaDjerada, ZoubirPréat, VéroniqueDjordjević, VericaPrestidge, Clive A.Djuris, JelenaPrévostel, CorinneDkhar, Hedwin KitdorlangPricl, SabrinaDługaszewska, JolantaPriola, EmanueleDobrzynski, PiotrPrivette Vinnedge, LisaDodi, GianinaProhens, RafelDodou, KalliopiPrzybyłek, MaciejDomb, AviPuchkova, Ludmila V.Dombrowski, YvonnePuglia, CarmeloDomínguez-Álvarez, EnriquePuiggalí-Jou, AnnaDonadio, GiulianaPunzo, FrancescoDong, WeibingPutra, Okky DwichandraDong, XiaoweiPutrinš, MartaDorlo, ThomasQi, FengDorta-Estremera, StephanieQi, ZhouDouroumis, DennisQin, XiaoliDrabczyk, AnnaQiu, MingfengDrăgoi, Cristina ManuelaQu, XiaozhongDreesen, ErwinQuagliariello, VincenzoDrolet, BenoîtQuan, GuilanDrozdov, Aleksey D.Quiñones, Luis AbelDroździk, MarekQuintanar-Guerrero, DavidDua, KamalQuintavalla, AriannaDuan, BinQuodbach, JulianDuan, XiaohuiR. El-Seedi, HeshamDuatti, AdrianoRabanal Anglada, FrancescDubbelboer, IlseRadchenko, Eugene V.Duconge, JorgeRadeghieri, AnnalisaDudhipala, NarendarRafael, DianaDugar, RohitRagno, GaetanoDugar, Rohit P.Ragusa, AndreaDuong, Van TuRahman, Khondaker MirazDuran Lobato, MatildeRai, AkhileshDurrant, Lindy G.Rai, AnkitDuskey, Jason ThomasRai, PrakashDutta, KingshukRaimi-Abraham, BahijjaDziadas, Mariusz TomaszRais, RanaDzimianski, JohnRajendran, Jagathesh ChandraDzmitruk, VolhaRajendran, Naresh KumarDzobo, KevinRajić, ZrinkaE Serda, RitaRallis, MichailEdginton, Andrea NRamalingam, PrakashEdwards, VinceRamasamy, ThiruganeshEedara, Basanth BabuRamos Cabrer, PedroEgan, Paul FRamsköld, DanielEgan, Paul F.Rancan, FiorenzaEgito, Eryvaldo Socrates Tabosa DoRanganathan, SrivathsanEgorova, KseniaRangger, ChristineEhrmann, AndreaRanjan, Amalendu P.Elaissari, AbdelhamidRanjan, AshishEleftheriadis, GeorgiosRapeanu, GabrielaEleonora, RussoRasskazov, IliaElfalleh, WalidRathinasabapathy, AnandharajanElhissi, AbdelbaryRau, IleanaElizalde, Luis E.Rauckhorst, AdamElmeshad, AliaaRautio, JarkkoEloy, Josimar De OliveiraRauwel, ProtimaEl-Say, Khalid MohamedRavaioli, StefanoElse, Terry AnnRavula, AbhigyanElShaer, AmrRegdon, GézaEndo, MakotoReis, CatarinaEnosi Tuipulotu, DanielReis, Catarina PintoEnrione, JavierReisfeld, BradErdem, EmreReissmann, SiegmundErdő, FranciskaReker, DanielEric Kast, RichardRemedios Serrano, DoloresErxleben, AndreaRen, YongEscargueil, AlexandreRengasamy, KannanEscoffre, Jean-MichelRenò, FilippoEsposito, ElisabettaRenu, SankarEsposito, Emilio XavierRenukuntla, JwalaEspuelas, SocorroRepka, MichaelEtrych, TomášReverter, EnricEudald, CasalsRho, Hoon SukEvans, CameronRiau, Andri KartasasmitaFacchetti, GiorgioRibeiro, AntonioFadda, Anna MariaRibeiro, HelenaFagioli, FrancaRibeiro, Helena MargaridaFais, StefanoRibeiro, Viviana P.Fakhraei Lahiji, ShayanRicci, MaurizioFakhrullin, Rawil F.Richard, CyrilleFalsini, SaraRichards, Dylan J.Faneca, HenriqueRichards, Dylan JackFang, JunRichardson, AlanFang, LiangRichardson, JosephFang, YiRinaldi, FedericaFang, Yi-PingRinnerthaler, MarkFanizza, ElisabettaRipoli, CristianFareed, JawedRistori, SandraFarhan, MohammadRizvanov, AlbertFariña, José B.Rizzi, RosannaFarkas, AttilaRizzo, FabioFasinu, PiusRizzolio, FlavioFatouros, Dimitrios G.Roberts, Arthur G.Feczko, TivadarRobinson, NirmalFeczkó, TivadarRocha, JoãoFederica, FogliettaRochfort, KeithFeitosa, EloiRodallec, AnneFelgueiras, HelenaRoderfeld, MartinFeng, Sheng-WeiRodilla, VicentFenyvesi, FerencRodrigues, JoãoFernandes, HugoRodriguez, LuisFernandes-Cunha, Gabriella MariaRodríguez-Hornedo, NaírFernández-Arévalo, MercedesRoggo, YvesFernandez-Campos, FranciscoRogobete, Alexandru FlorinFernandez-Garcia, RaquelRom, SlavaFernández-Gutiérrez, MarRomano, Giovanni LucaFerraz Gonçalves, José AntónioRomański, MichałFerreon, Allan Chris M.Rombolà, LauraFerriz-Martínez, Roberto AugustoRomerio, FabioFichera, EpifanioRomero-Uribe, GabrielaFierascu, Radu ClaudiuRomiti, Giulio FrancescoFigueiras, AnaRonsard, LaranceFigueiredo, Isabel VitóriaRoohnikan, MahdiFigueiredo, Marxa L.Rosa, Arianna CarolinaFilho, Marcílio CunhaRosado, CatarinaFilice, MarcoRosales-Hernández, Martha CeciliaFilippov, SergeyRosania, GustavoFilova, EvaRoseanu, AncaFini, PaolaRosenholm, JesiccaFinke, Jan HenrikRoseti, LiviaFinsterer, JosefRosière, RémiFiorica, CalogeroRossi, AlessandraFirdessa, RebumaRossi, FilomenaFitzgerald, SeánRossi, SilviaFlament, Marie-PierreRosso, NataliaFlorczyk, StephanieRoszkowska, AnnaFlores, Ana IsabelRothe, ThomasFocaroli, StefanoRotondo, John CahrlesFogacci, FedericaRotstein, ColemanFontana, AntonellaRoullin, V. GaëlleFonte, PedroRozsival, PavelFORGET, PATRICERozza, ArianeForini, Francesca S.Rubilotta, EmanueleFornaguera, CristinaRubino, IlariaFortuna, AnaRubio Alonso, JuanForzato, CristinaRuda-Kucerova, JanaFracasso, GiulioRudd, Sean GFraguas-Sánchez, Ana IsabelRudra, ArnabFraix, AuroreRuiz, AmaliaFrancesco, ScavelloRuiz-Caro, RobertoFranco, Marina SantiagoRuiz-Ederra, JavierFrancuzik, WojciechRusciano, DarioFrandsen, Stine KrogRusso, PaolaFrank, LuizaRuzza, ChiaraFrank, Luiza AbrahãoRybarczyk-Pirek, Agnieszka J.Franzè, SilviaRybka, Jakub DaliborFreisleben, Hans-JoachimRychahou, PiotrFreitas, Vanessa MoraisRyu, Ji HyunFrey, KathleenRyu, Jung SuFrizzo, Clarissa P.Sa-Barreto, Livia L.Froelich, AnnaSabatino, DavidFröhlich, EleonoreSachar, MadhavFrolov, Andrey I.Sacher, StephanFromen, CathySack, UlrichFu, JunjieSah, HongKeeFuhrmann, GregorSahota, Tarsem S.Fujii, MakikoSaint-Pol, JulienFujinaga, KohSalamanca, Constain H.Fukuta, TatsuyaSalameh, ChrystelleFumoto, ShintaroSalaün, FabienFurlani, FrancoSaleem, ImranFurneri, Pio MariaSaleh, TawfikFuruishi, TakayukiSalhab, HassanGabrielli, ValeriaSaliev, TimurGabrielson, Nathan P.Salmina, AllaGadjanski, IvanaSalunke, SmitaGadomska-Gajadhur, AgnieszkaSamal, SangramGaffet, EricSamanta, SubarnaGala, Rikhav P.Sambuy, YulaGalateanu, BiancaSamson, Abraham O.Galbis, ElsaSánchez Navarro, AmparoGalderisi, UmbertoSanchez, ElenaGalelli, LucaSánchez-García, DavidGallardo, EugéniaSandoval-Yáñez, ClaudiaGallo, JuanSandri, GiuseppinaGalluccio, MicheleSands, JeffGalstyan, AnzhelaSansone, FrancescaGaman, Mihnea-AlexandruSanti, MelissaGanazzoli, FabioSanti, PatriziaGanesan, A.Santos, Adriana O.Gangadaran, PrakashSantos, Cleydson Breno R.Ganguly, SamitSantos, MiguelGao, ChunshengSantos-Coquillat, AnaGao, HuileSanz-Clemente, AntonioGao, JinSapino, SimonaGao, ZheSapudom, JiranuwatGarcia-Lopez, PatriciaSara, ZalbaGarcía-Martínez, EvaSaravanakumar, KandasamyGarcía-Mena, JaimeSarli, VasilikiGarcía-Ramos, Juan CarlosSarraguça, MafaldaGarcía-Villén, FátimaSarsour, EhabGarello, FrancescaSasai, YasushiGarrido, María JesusŠatkauskas, SauliusGarriga, RosaSato, HideyukiGashti, Mazeyar ParvinzadehSato, KazuhideGaspar, Maria ManuelaSato, ShintaroGáspár, RóbertSato, ShuzoGatzka, MartinaSattler, WolfgangGAUTHIER, PASCALESautto, Giuseppe A.Gaviña, PabloSawada, KazuhikoGe, JiaojuSayers, Edward JGelfuso, Guilherme M.Sazonova, Margarita A.Gendaszewska-Darmach, EdytaŠčasný, MilanGenta, IdaSchaiquevich, PaulaGeraci, FabianaSchlereth, Simona L.Gerogiorgis, DimitriosSchlich, MicheleGeszke-Moritz, MałgorzataSchlindwein, WalkiriaGeyer, JoachimSchmidl, DoreenGhadiri, MalihehSchmutz, MarcGhazanfari, LidaSchnieke, Angelika E.Ghersi, GiulioSchols, DominiqueGhica, Mihaela VioletaScholz, CarmenGhica, Mihaela-VioletaSchönthal, Axel H.Ghiciuc, CristinaSchulz, FlorianGhiciuc, Cristina MihaelaSchumann, CananGholamipour-Shirazi, AzarmidokhtSchwaminger, SebastianGhonaim, HassanScognamiglio, Pasqualina LianaGhori, Muhammad UsmanScott, ChristopherGhosh, SantoshSegale, LorenaGiansanti, LuisaSeifert, GotthardGigliotti, CasimiroSeiler, Magdalene J.Gikas, EvagelosSelbo, Pål KristianGil, Jesus PerezSelin, VictorGill, JasonSelwood, David L.Gillespie, James W.Seo, Ho SeongGiovagnoli, StefanoSerda, MaciejGiovannelli, PiaSerpe, LoredanaGiovannoni, Maria P.Serrano, Dolores R.Gisbert-Garzarán, MiguelSeto, YoshikiGitlin-Domagalska, AgataSettimio, PacelliGitsov, IvanSgarbi, GianlucaGiunchedi, PaoloShabatina, TatyanaGlaser, KeithShah, Dhaval K.Glass, JonShahjamali, MohammadGlassman, Patrick M.Shaik, Anver BashaGlišić, BiljanaShakeel, FaiyazGloria, AntonioShakibaei, MehdiGlushakov, AlexanderShakya, AkhileshGoins, William F.Shamieh, SaidGok, OzgulShankar, EswarGołda-Cępa, MonikaSharbeen, GeorgeGomes-da-Silva, Lígia C.Shariare, MohammadGomez Cerezo, Maria NatividadSharma, PrashantGomez-Gil, VeronicaShavandi, AminGomez-Ruiz, SantiagoShcharbin, DzmitryGonçalves, LídiaSheikh Mohamed, M.Gonzalez Alvarez, IsabelShen, Hsin-HuiGonzález Laredo, Rubén F.Shen, JieGonzález Maglio, Daniel H.Shen, ZhiqiangGonzalez, Jimena S.Sheng, RuilongGonzález, MartaShevtsov, MaximGonzalez-Alvarez, IsabelShi, LingyanGonzalez-Alvarez, MartaShi, WenGonzález-Nieto, DanielShibli, Jamil AwadGopalakrishnan, GopakumarShimizu, TaroGordeeva, OlgaShimizu, TatsuyaGoreham, ReneeShimodaira, ShigetakaGorman, GregShimpi, ManishkumarGorovits, BorisShin, Beom SooGorzelanny, ChristianShin, Crystal S.Gośliński, TomaszShin, Meong CheolGosselin, RyanShin, SoyoungGoto, MasahiroShirasaka, YoshiyukiGoto, RiekoShirazian, SaeedGoudoulas, ThomasShugo, SuzukiGoyanes, AlvaroShukla, Snehal K.Gozalbes, RafaelSi, KeGracia-Sancho, JordiSiafaka, PanoraiaGradzielski, MichaelSiamidi, AngelikiGrande, RossellaSideratou, ZiliGrassi, MarioSidorenko, Viktoriya S.Grazu, ValeriaSidoti, AntoninaGreenhalgh, DavidSiegmund, WernerGrégoire, SébastienSieni, ElisabettaGrice, Jeffrey E.Signore, GiovanniGriffin, Brendan T.Sikora, MariuszGrijalvo, SantiagoSilva Filho, EdsonGrimaudo, Maria AuroraSilva, Ana CatarinaGrivas, IoannisSilva, JoanaGrivel, Jean-ClaudeSilva, Nuno Helder Da Cruz SimõesGrossi, GiancarloSilva, SofiaGrumezescu, Alexandru MihaiSilva-Abreu, MarcelleGrzeskowiak, BartoszSilveira, Nádya P. DaGudey, Shyam KumarSilvestri, BrigidaGuerrero-Martínez, AndrésSimeone, PasqualeGugliandolo, EnricoSimoes, SandraGuillaume, OlivierSimon, LaurentGuiot, CaterinaSingaraju, AdityaGukasyan, HovikSingh, Ajay VikramGulei, DianaSingh, IshwarGumieniczek, AnnaSingh, MogieGun’ko, Vladimir MSinha, AbhijeetGunasekar, Susheel K.Sinko, PatrickGuo, BaolinSiracusa, RosalbaGuo, Chih-HungSitar, Daniel S.Guo, DandanSivalingam, PeriyasamyGupta, PrachiSkalko-Basnet, NatasaGupta, SanjaySkerra, ArneGustafsson, Mats G.Šklubalová, ZdeňkaGutierrez-Aguilar, ManuelSkotnicki, MarcinGuzmán-Navarro, ManuelSkouras, AthanasiosGuzow-Krzemińska, BeataSlimano, FlorianHa, Eun-SolSloan, Alastair J.Haas, HeinrichSmaldone, Gerald C.Haddrell, Allen E.Smani, YounesHadinoto, KunnSmarandache, AdrianaHaemmerich, DieterŚmiga-Matuszowicz, MonikaHageman, MichaelSmith Callahan, Laura A.Hagenbuch, BrunoSmit-Mcbride, ZeljkaHagesaether, EllenSnežana, PapovićHama, SusumuSoares, Graca M.B.Hamman, JoiasSobczak, MarcinHan, FelicitySoheila, Ali Akbari GhavimiHan, SifeiSoica, CodrutaHan, XiuguoSolimando, Antonio GiovanniHan, YingchaoSolomon, MelaniHanafy, Nemany A. N.Solopov, PavelHanawa, TakehisaSolorio, LuisHandral, HarishSong, ChunhuaHanieh, Patrizia NadiaSong, Im SookHann, Hie-WonSong, JianxunHansen, NielsSong, LiujiangHanson, BenSong, LushengHao, JinsongSong, XinhuaHardies, Stephen C.Song, ZiyuanHardtke-Wolenski, MatthiasSorrenti, MilenaHardy, John G.Sotiriou, GeorgiosHarker, AnthonySoto, FernandoHaron, MonaSou, KeitaroHasan, AbsharSoulairol, IanHasanzadeh Kafshgari, MortezaSour, AngéliqueHasegawa, UraraSovány, TamásHashidzume, AkihitoSovinco, FabioHashimoto, MichinaoSpanakis, MariosHatahet, TaherSpartalis, EleftheriosHathout, RaniaSpetea, MarianaHathout, Rania MohammedSpingler, BernhardHatziantoniou, SophiaSpoerk, MartinHaware, Rahul V.Squadrito, Mario LeonardoHawcutt, Daniel B.Squassina, AlessioHawthorne, SusanSrcic, StankoHayashida, MorihiroSreedharan, SreejeshHe, HongliangSreerama, SubramanyaHe, JingStacchiotti, AlessandraHe, MeiStamm, ManfredHe, MingyuStanek, AgataHe, WeilueStankov, KarmenHealy, Anne MarieStarynowicz, PrzemyslawHeard, CharlesStavros, MalamatarisHeath, TimothyStawny, MaciejHeger, ZbynĕkSteenekamp, JanHeiduschka, PeterStefanucci, AzzurraHeinämäki, Jyrki TapioSteiner, DeniseHellstrom, PerŠtěpánek, F.Hémadi, MiryanaStepanenko, Aleksei A.Hendriks, Hans H.Stepensky, DavidHens, BartStephan, HolgerHering-Smith, KathleenSteup, MartinHerraez, ElisaStieger, BrunoHertzler, RussellStobiecka, MagdalenaHeskamp, SandraStoddard, ShanaHessel-Pras, StefanieStolp, HelenHildreth, Blake E.Stratford, Robert E.Hilgendorf, ConstanzeStrickley, RobertHinrichs, Wouter L. J.Stringaro, AnnaritaHiratsuka, MasahiroSu, ChangqingHirayama, FumitoshiSu, Chie-ShaanHiroaki, HidekazuSu, YeuHirose, RyoheiSu, Zheng-YuanHirschberg, CosimaSuarato, GiuliaHo, Tsing-FenSubhan, Md AbdusHoeben, RobSuda, KenichiHoffmann, Klaus H.Suga, HirotakaHofmann, UteSugano, KiyohikoHollmann, AxelSugimoto, HiroshiHolst, HelleSugiyama, HirokazuHomem-de-Mello, MauricioSuh, Hyung JooHooper, Douglas C.Sui, YongjunHorhat, Florin GeorgeSun, CaijunHori, AkikoSun, ConroyHornebecq, VirginieSun, HaoHorowitz, John D.Sun, JiazhenHortelano, GonzaloSun, JunjiangHoskins, ClareSun, LemingHossain, A. S. M. Monjur AlSun, ZhixiongHoward, Erin W.Sundararajan, RajiHowes, PhilipSuntornnond, RatimaHsia, Shih-MinSur, SubhayanHu, JackSuresh, KuthuruHu, Shang-HsiuSutariya, Vijaykumar B.Hu, Teh-MinSuzuki, Jon B.Hu, XianglongSuzuki, ToyofumiHua, Kuo-FengSwindle, MichaelHua, XiaoqingSyed, AsadHuang, JiangengSykłowska-Baranek, KatarzynaHuang, WeiSytar, OksanaHuang, YuanyuSzczepanowicz, KrzysztofHuang, YugangSzékely, AndreaHuang, YunlongSzeleszczuk, ŁukaszHuerta-Angeles, GloriaSzewczyk, Nathaniel J.Huhtinen, Mirja K.Szlęk, JakubHui, Patrick C.L.Szulc-Dąbrowska, LidiaHuntosova, VeronikaSzwajca, AnnaHurtig, MarkSzweda, PiotrHutchins, Kristin M.Szymańska, EmiliaHuth, SebastianTacconelli, StefaniaHwang, MintaiTachibanaki, ShujiHwang, Seung RimTadić, VanjaHwang, Sung-JooTagad, HarichandraIaccino, EnricoTagit, OyaIacone, RobertoTaglietti, AngeloIannuccelli, ValentinaTai, WanboIatridi, ZacharoulaTajber, LidiaIatrou, HermisTakacs-Novak, KrisztinaIbrahim, Sherif AbdelazizTakács-Novák, KrisztinaIbrahim, ToniTakahara, MariIglesias, BernardoTakatani-Nakase, TomokaIgnat, MariaTallapaka, Shailendra B.Ignjatovic, NenadTallarico, MarcoIkushiro, Shin-ichiTambuwala, MurtazaIlaria, OrtensiaTamošiūnas, Algirdas EdvardasIlhan, HarunTampa, MirceaIlic-Stojanovic, SnezanaTampucci, SilviaIlies, Marc A.Tan, SongwenIlyinskii, Petr O.Tan, XuIm, Chang-NimTanaka, TakujiImberti, CinziaTanaka, TomonariImperatore, RobertaTanc, MuhammetInbraj, StephenTang, JingInfante, ArantzaTang, YuanIngelsson, MartinTaranto, Alex GutterresInnamorati, GiulioTatischeff, IrèneInoue, DaisukeTatu, Alin LaurenţiuIonescu, Mihaela IleanaTaurin, SebastienIonita, PetreTavakoli, JavadIorio, Ronald M.Tavares, AnthonyIqbal, Hafiz. M. NTaylor, Rebecca E.Iqbal, ShoaibTekko, IsmaielIrie, TetsumiTeleki, AlexandraIrwin, David C.Terao, JunjiIsola, GaetanoThackaberry, Evan A.Israel, Liron LimorThaker, Youg RajIsreb, AbdullahThakur, Basant KumarIssa, Michele GeorgesThakur, SachinIta, KevinTheile, DirkIto, ShingoThommes, MarkusIwasaki, TakashiTian, BoIwasaki, YoshiakiTian, JiangweiJ. Storkus, WalterTian, JustinJagusiak, AnnaTien, An-ChiJain, AkshayTiera, MarcioJaiswal, Jagdish KumarTikoo, Suresh K.Jaiswal, Jyoti K.Timmins, PeterJakimovski, DejanTing, Jeffrey M.Jakubowski, WitoldTiwari, RoshanJammalamadaka, UdayabhanuTodorov, Svetoslav DimitrovJamwal, RohitashTogami, KoheiJanagam, Dileep R.Tokars, Valerie L.Janagam, Dileep ReddyTomatsu, ShunjiJanek, TomaszTomić, SimonidaJang, Keon-SooTomlin, Stephen R.Jang, Mi-KyeongTomuta, IoanJanicka, MałgorzataTong, ZhenboJanjic, Jelena M.Topuz, FuatJanoušková, OlgaTorrado, Juan J.Jaramillo-Flores, MariaTorrado-Santiago, SantiagoJaromin, AnnaTorre, Maria LuisaJasminder, SahiTorres-Suárez, Ana-IsabelJaszcz, KatarzynaTosi, GiovanniJedelská, JarmilaTouraud, DidierJee, Jun-PilToutain, Pierre LouisJee, ShiouhwaTran, FabienJelonek, KatarzynaTran, PhuongJelsch, ChristianTran, TTDJencova, VeraTrandafirescu, Cristina-MariaJenkins, Samir V.Trapani, IvanaJensen, Svend BorupTricarico, DomenicoJeon, JonghoTripodo, GiuseppeJeong, Hyun YoungTrivedi, VivekJeong, Jun-hoTroutman, Jerry M.Jeong, Seong HoonTrucillo, PaoloJiang, ChengminTrujillo De Santiago, GrisselJiang, YanbinTrujillo-Cayado, LuisJiang, ZiwenTruong, Nghia P.Jianliang, ShenTruong, Vi-KhanhJicsinszky, LászlóTsapis, NicolasJin, Jun-OTsiourvas, DimitriosJin, SunggiuTsiourvas, DimitrisJin, XinTsirigotis-Maniecka, MartaJindřich, JindřichTsukamoto, TetsuoJing, HuiTsuneoka, MakotoJo, Yeon-JiTu, Ting-YuanJones, MarjorieTupally, Karnaker R.Joshi, AdityaTurcan, SevinJoshi, SameerTyliszczak, BożenaJović, DanicaTyurin-Kuzmin, PyotrJoyce, PaulUchida, SatoshiJózefczak, ArkadiuszUeng, Yune-FangJuang, Yi-JeUhrikova, DanielaJuarez, KarlaUlbert, SebastianJuhasz, KataUmerska, AnitaJuif, Pierre EricUngaro, FrancescaJuluri, AbhishekUngurianu, AncaJung, HyungilUpadhya, Manoj AtmaramjiJung, Jae HwanUppulury, KarthikJung, SeunhoUrbančič, IztokJung, YunjinUrbanczyk-Lipkowska, ZofiaJungsuwadee, PaiboonUrbanelli, LorenaJunsong, LiUritu, Cristina MarianaJuppo, AnneUrschitz, JohannKabała-Dzik, AgataUrso, ElenaKachrimanis, KyriakosUrtti, ArtoKaczmarek, BeataUthaman, SajiKadam, AvinashUzbekov, Rustem E.Kalhapure, Rahul S.Vaidya, AmitaKalia, Yogeshvar N.Vaidya, BhuvaneshwarKalló, GergőVaidyanathan, GanesanKalyan, RameshVaidyanathan, SriramKamada, KaiValable, SamuelKambayashi, AtsushiValente, Artur J. M.Kambhampati, Siva PramodhValsami, GeorgiaKamihira, MasamichiVamanu, EmanuelKaminski, PiotrVan Den Mooter, GuyKamiński, TomaszVan Riet-Nales, Diana A.Kanada, MasamitsuVanaki, ShayanKang, Han ChangVance, DavidKang, Hee EunVandamme, ThierryKang, Myung JooVangala, AnilKang, Nae GyuVangala, VenuKang, Yunqing (Kevin)Vanic, ZeljkaKankala, Ranjith KumarVanić, ZeljkaKanugula, Anantha KoteswararaoVaquero, JavierKao, Chai-LinVáradi, CsabaKao, Li-TingVáradi, JuditKaralis, Vangelis D.Varga, ZoltanKaramouzis, MichalisVarna, MarianaKarashima, MasatoshiVarum, FelipeKarazniewicz, MartaVashist, ArtiKarbownik, AgnieszkaVasic, VesnaKarcz, DariuszVasiliu, SilviaKardas, PrzemyslawVazquez, EstherKarewicz, AnnaVázquez-López, Ezequiel M.Karim, NasiaraVedagopuram, SreekanthKaruppan, Mohan Kumar MuthuVée, Marc LeKasperczyk, JanuszVeeravalli, VijayabhaskarKasperek, ReginaVel, Bharath KumarKasprzak, ArturVelasco, Rebeca HernandezKastellorizios, MichailVelikova, TsvetelinaKatarzyna, MalarzVelikyan, IrinaKathuria, HimanshuVemulapalli, VidyasiriKatona, GáborVenkatadri, RajkumarKaushik, NehaVenkatesan, JayachandranKauss, TinaVentura, Cinzia AnnaKawakami, KohsakuVeréb, Zoltán JánosKawakami, ShigeruVergara, DanieleKawano, KumiVergnes, BrunoKazakov, SergeyVerron, EliseKelarakis, AntoniosVeszelka, SzilviaKepinska, MartaVetvicka, VaclavKerr, IanVida, YolandaKertmen, AhmetVidal, FernandoKesharwani, PrashantVíglaský, ViktorKesharwani, SiddharthVijayakumar, SekarKesharwani, Siddharth S.Vilella, AntoniettaKhalf, AbdurizzaghVillarreal-Gómez, Luis JesúsKhan, Omar F.Vilsinski, Bruno HenriqueKhandavilli, UdayaVinarov, ZahariKhilwani, RakeshVine, Kara L.Khoder, MouhamadVingolo, Enzo MariaKhudyakov, Igor VVitale, FabrizioKhung, Yit LungVitorino, CarlaKhurana, VarunVivas Mejia, PabloKhurshid, ZohaibVivero-Escoto, JuanKhutoryanskiy, VitaliyVives, JoaquimKijenska, EwaVlahovic, TraceyKim, Dong WukVlaia, LaviniaKim, Il WonVlase, GabrielaKim, Jeong-HwanVlase, TitusKim, Jin-SeokVogelbaum, Michael A.Kim, JinwookVoliani, ValerioKim, Keun-SikVora, LalitkumarKim, Ki-TaekVorobyeva, AnzhelikaKim, KwanohVoshavar, ChandrashekharKim, Min-SooVostinaru, OliviuKim, Ok-KyungVrečer, FrancKim, Seong-GonVuddanda, Parameswara RaoKim, Sung-Kun (Sean)Vukmirovic, SasaKim, Yeu-ChunVulto, Arnold G.Kinaret, PiaVuorimaa-Laukkanen, ElinaKirby, DanielWaczulíková, IvetaKirton, Stewart B.Wagner, Karl G.Kiselev, AntonWalter, Fruzsina R.Kittel, AgnesWang, ChenguangKitzmüller, ClaudiaWang, FukeKlajnert-Maculewicz, BarbaraWang, FuyiKlang, VictoriaWang, LeiKlar, AgnesWang, LingleKleinebudde, PeterWang, PengchengKluczyk, AlicjaWang, QunKnapik-Kowalczuk, JustynaWang, Shi-BinKnezevic, NikolaWang, TaoKo, Eun-juWang, WeiKobuchi, ShinjiWang, WenxinKodidela, SunithaWang, XiaodongKoga, YoshikatsuWang, ZhenpingKöhler, KarstenWang, ZhijunKojima, ChieWang, ZhisongKolanjiyil, Arun VargheseWang, ZongjieKolar, MitjaWarnken, ZacharyKolisek, MartinWarzecha, ZygmuntKolmar, HaraldWatts, AlanKolobov, Alexandr AlexandrovichWei, GangKołodziejczyk-Czepas, JoannaWei, HuaKolosnjaj-Tabi, JelenaWei, Kai-CheKondo, HiromuWeighardt, HeikeKong, L.X.Weiss, Clemens K.König, JörgWeiss, JohannaKonjufca, VjollcaWeitschies, WernerKonstantinov, Spiro M.Weng, Yung-JinKontogiannidou, EleniWertz, Philip W.Kontoravdi, CleoWesołowski, MarekKoo, Hee-beomWestergaard, NielsKoolivand, AbdollahWibowo, DavidKootala, SujitWiener, ZoltánKopecki, ZlatkoWietrzyk, JoannaKopel, PavelWiglusz, Rafał JakubKorchowiec, BeataWildemann, BrittKorkmaz, EmrullahWilliams, GarethKoroleva, Marina YuWilliams, Noelle S.Korupalli, ChiranjeeviWilner, Samantha E.Korzhikova-Vlakh, EvgeniaWinnicka, KatarzynaKorzhikov-Vlakh, ViktorWitkowska Nery, EmiliaKosicka-Noworzyń, KatarzynaWojciechowski, KamilKostić, Aleksandar Ž.Wölk, ChristianKostka, LiborWołowiec, StanisławKotlyarova, AnastasiiaWong, Boon SengKotyla, Przemyslaw J.Wong, Chin-CheanKovacs, AnitaWong, Dennis CCKozak, MaciejWoźniak-Budych, MartaKozielski, LucjanWright, DavidKoziolek, MirkoWu, HuanKozłowska, JoannaWu, JunKozlowska, JustynaWu, WeiKragović, MilanWu, Yu-TseKrajczewski, JanWu, ZhangxiongKrasnikov, BorisWurm, FlorianKrishnan, VinuWuttke, StefanKruger, CherieXi, GuifaKrüger, MarcusXia, TianKrupa, AnnaXiao, ChunshengKubiak, KatarzynaXing, GengmeiKubo, Anna-LiisaXiong, JiaKubo, YoshiharuXiong, ZhaohuiKucuk, IsrafilXu, CancanKuddannaya, ShreyasXu, LuKuehl, Philip J.Xu, PengfeiKuentz, MartinXu, RongKulbacka, JulitaXu, TaoKulkarni, KetavXu, Xiao-DingKumar K. B., VinayaXu, Yan-MingKumar Patel, SravanXue, JiajiaKumar, JananiXue, MingKumar, MukeshYadavalli, TejabhiramKumar, PradeepYAKAVETS, IlyaKumar, RajYallapu, MuraliKumar, RameshYallapu, Murali MohanKumar, SamirYan, DayunKumar, VineetYan, LifengKumeria, TusharYan, XuehaiKunda, NiteshYaneva, Zvezdelina L.Kung, Chin-PingYang, Chih-TsungKuo, Yao-HaurYang, Chul-SuKurek, MateuszYang, ChunhuaKurganov, BorisYang, FangKurrikoff, KaidoYang, FengqingKusamori, KosukeYang, Hung-WeiKushwah, VarunYang, JianminKwiatkowski, PawelYang, Kai-ChiangKwilas, StevenYang, Seung YunKwok, Henry Hang FaiYang, XiaochunKwon, Il-KeunYang, YannanKwon, Tae-HyukYang, YongkangKwon, Young M.Yaoita, YasunoriLadyzynski, PiotrYapp, Donald T.Lahiji, Shayan FakhraeiYaraki, Mohammad TavakkoliLahiri, TanayaYasuda, TakakoLai, Chian-HuiYasunaga, MasahiroLai, FrancescoYazdi, ImanLai, Jui-YangYazdimamaghani, MostafaLakshminarayanan, RajamaniYen, Meng-ChiLalatsa, AikateriniYeo, SynLalic-Popovic, MladenaYi, TaoLangner, MarekYildirim, AdemLankoff, AnnaYilmaz, Catalina Natalia CheaburuLannoy, DamienYilmaz, MehmetLanza, MarcoYin, GuoweiLanza, MicheleYin, JunLapteva, MariaYokota, Shin-ichiLaquintana, ValentinoYokoyama, ShinjiLargent-Milnes, TallyYonemochi, EtsuoLarhrib, HassanYong, Kar WeyLasagni, LauraYongxiang, LuoLatif, MuhammadYoo, Hyuk SangLau, Wai Man RaymondYook, SimmyungLaurini, ErikYoon, Bo-EunLauro, Maria RosariaYoon, In SooLauzon, Marc-AntoineYoshida, KentaroLazzara, GiuseppeYoshii, HidefumiLe Gall, TonyYoshioka, Ken-ichiLe, Hoang-ThanhYounes, HusamLebeau, LucYoung, Howard A.Lebrón, José AntonioYu, Deng-GuangLeclair, GregoireYu, LuLeclerc, DenisYu, Weiling (Connie)Lee, AejuYuan, Wei-EnLee, A-WYuan, ZhihongLee, Beom-JinYuan, ZhiqinLee, Chia-HungYuba, EijiLee, Heung KyuYue, PengfeiLee, Hong-KiYumura, ShigehikoLee, Hye SukYutani, ReikoLee, JaehwiYu-Wai-Man, CynthiaLee, Jangik IkeZabielska-Koczywąs, KatarzynaLee, JeongmiZaboronok, AlexanderLee, Jong BongZaccheroni, NelsiLee, Jung SeokZahari, VinarovLee, SangkilZahid, MalihaLee, TuZaid, Abdel NaserLee, WonhyeZaman, FarasatLee, Yi-ChihZambito, YleniaLee, YongbokZamostny, PetrLee, YonghyunZamparini, FaustoLEGRAND, François-XavierZapotoczny, SzczepanLehr, ThorstenZarzuelo Castañeda, AránzazuLei, WeiZarzycki, Pawel K.Leitner, Wolfgang WZdarta, AgataLemmerer, AndreasZdarta, JakubLeporatti, StefanoZelkó, RománaLeppert, WojciechZeng, BijunLesnikowski, Zbigniew J.Zeni, OlgaLesuisse, DominiqueZhang, DianbaoLesyk, Roman B.Zhang, HongboLeung, Ada W.Y.Zhang, HuijieLeung, Shui YeeZhang, JinjinLevina, AvivaZhang, LiqiangLewandowska, KatarzynaZhang, RujingLewis, GladiusZhang, TianyuLeyssens, TomZhang, WeizhongLeyva-Gómez, GerardoZhang, XiaoyuLi, ChuanZhang, XingminLi, FengZhang, XuanLi, FuhaiZhang, XunliLi, GuangdiZhang, YajieLi, JasonZhang, YiLi, JieZhang, YuLi, JinyaoZhang, YueLi, JunjieZhang, YufengLi, LiZhang, YumiaoLi, LipingZhao, ChunxiaLi, MengZhao, MinLi, NaZhao, QinjianLi, S. KevinZhao, XiuhuaLi, Wen-WuZhao, YuanliLi, XiaohongZhao, ZhenjiangLi, XueZheng, XintingLi, YangZhou, DongfangLi, YunfeiZhou, DongmingLi, ZhiyuZhou, JichunLiao, Ai-HoZhou, KunLiao, YonghongZhou, QunLibra, MassimoZhou, YubinLietzau, GrazynaZhou, ZhuxianLim, Eun-KyungZhu, CaihongLim, JasonZhu, ChunleiLima, SofiaZhu, HongyanLin, HaishuZhu, JingenLin, Hai-ShuZhu, LanLin, Hsiu-MeiZhu, LiminLin, Peter P.Zhu, YishenLin, T. HZiaee, AhmadLin, Tung-YiZidek, JanLin, Wei-ChaoZiegler-Borowska, MartaLin, WeifengZiemssen, FockeLin, WenjingZingg, Jean-MarcLin, YangZmudzki, PawelLin, ZhoumengZobi, FabioLing, ChenŻołek, TeresaLipke, PeterZorov, Dmitry BLis Arias, Manuel J.Zu, YouliLiu, Chao-LinZuber, GuyLiu, Cheuk LunZuccari, GuendalinaLiu, Der-zenŽugić, AnaLiu, FangZúñiga, LeandroLiu, FeiZurth, ChristianLiu, LiZvonar, AlenkaLiu, Ting-YuZydowsky, Thomas MLiu, YuZyuzin, Mikhail V.

